# Nutritional Status and Feeding Regimen of Patients with Esophagus Cancer—A Study from Vietnam

**DOI:** 10.3390/healthcare9030289

**Published:** 2021-03-06

**Authors:** Binh Pham Van, Hoa Nguyen Thi Thanh, Huong Le Thi, Anh Nguyen Le Tuan, Hang Dang Thi Thu, Dung Dang Viet

**Affiliations:** 1 Abdominal Surgery Department 1 and Robotic Surgery Center, Vietnam National Cancer Hospital, Hanoi 12511, Vietnam; 2 Clinical Nutrition Center, Vietnam National Cancer Hospital, Hanoi 12511, Vietnam; dr.peace2801@gmail.com (H.N.T.T.); Danghang271295@gmail.com (H.D.T.T.); 3 Institute of Preventive Medicine and Public Health, Hanoi Medical University, Hanoi 11521, Vietnam; dennguyenle@gmail.com; 4 Gastrointestinal Surgery Center, 103 Military Hospital, Military Medical Academy, Hanoi 12109, Vietnam; Dungb2103@gmail.com

**Keywords:** esophagus cancer, nutrition status, dietary intake, malnutrition

## Abstract

Background: Esophagus cancer patients are at high risk of malnutrition. This study was performed to assess the nutritional status and dietary intake of newly diagnosed esophageal cancer patients in Vietnam National Cancer Hospital (NCH). Methods: A cross-sectional study was conducted on 206 early esophageal cancer inpatients after gastrostomy from September 2017 to June 2018. The chi-squared test, Fisher exact test, and Mann–Whitney test were performed. The software of the Vietnam National Institute of Nutrition was used to evaluate the dietary intake of patients. Results: All the participants were male with a mean age of 57.1 ± 8.5 years. Overall, 87.4% of patients had dysphagia. Furthermore, 82.5% and 90.8% of patients reported weight loss one and six months pre-diagnosis, respectively. Moreover, 52.9% of patients suffered from mild/moderate malnutrition and 29.6% of patients had severe malnutrition according to the Patient-Generated Subjective Global Assessment (PG-SGA). The body mass index (BMI) and mid upper arm circumference (MUAC) measurement revealed 47.6% and 50% of undernourished patients, respectively. The proportions of patients having malnutrition were 10.7%, 55.8%, and 27.2% according to albumin, prealbumin, and total lymphocyte counts, respectively. The means of energy, protein, lipid, and carbohydrate in the patients’ 24 h preoperative diets were 973.6 ± 443.0 kcal/day, 42.4 ± 21.6 g/day, 31.0 ± 15.5 g/day, and 130.0 ± 64.5 g/day. The total energy, total protein, animal protein, total lipid, and plant lipid in the dietary intake of patients were strongly correlated with age, economic classification, and PG-SGA (each *p* < 0.05). The total energy intake increased day by day, with the average energy intake of 1343.9 ± 521.3 kcal on the seventh day. Energy and protein response rates increased day by day and were highest at 7 days post-operation at 18.0% and 19.4%. Conclusion: Malnutrition and insufficient intake are noteworthy in esophageal cancer patients. The PG-SGA is strongly correlated with the dietary intake of patients. The results from this study will help medical staff to prevent malnutrition and improve the nutritional status of esophageal cancer inpatients. Furthermore, public awareness should be raised on recognizing weight loss as an early symptom of esophageal cancer and the utilization of preoperative assessment tools for nutritional assessment and malnutrition management.

## 1. Introduction

Esophageal cancer is the seventh-highest cancer incidence and the sixth most common cause of cancer mortality in the world [1]. It is also the sixth most common cancer in men in Vietnam and seventh in the world [2]. It affected more than 600,000 people worldwide in 2020 [3] and is amongst the deadliest cancers with a 5-year survival rate of only around 17% [4,5]. The incidence of esophageal cancer is rapidly increasing. From 1990 to 2017, the global number of new esophageal cancer cases increased by more than 50% [6]. In England and Wales, the age-standardized incidence has increased by almost five times in both men and women from 1971 to 2001 [7]. In the same period of time, the incidence rate of esophageal adenocarcinoma, one of the main two types of esophageal cancer along with squamous-cell carcinoma [5], had the fastest increase, more than any other major cancers in the USA [8]. In 2017, this cancer caused almost 10 million disability-adjusted life years (DALYs) worldwide [6].

Cancer patients are at higher risk of malnourishment as it is associated with complications caused by cancer, such as treatment toxicity, inadequate food intake, reduction of physical functioning, etc. [9,10,11]. It was estimated that 20% to more than 70% of the total cancer patients worldwide suffered from malnutrition, and the figures varied amongst different types of cancer and patients’ ages [12]. Patients with cancers related to the gastrointestinal tract are also at substantially high risk of malnutrition [12,13]. It could be due to the blockages and interference with the food flow of the tumors that caused dysphagia to the patients [14]. Studies of 154 French hospital wards showed that up to 60.2% of patients with esophageal and/or gastric cancer suffered from malnutrition [15]. A study of patients with upper digestive tract cancer in a hospital in Spain found that more than half of them suffered from weight loss and 36.8% of the patients with weight loss lost more than 10% of their total weight [16]. A hospital-based cohort study in the Netherlands reported that 17% of esophageal cancer patients lost more than 10% of their usual weight within 3 months before the cancer notification [17]. In Vietnam, a retrospective cohort study in Ho Chi Minh City on 459 patients with gastrointestinal cancer reported a malnutrition prevalence of 19% based on the measurement of body mass index (BMI) and serum albumin level. Another cross-sectional study in Hanoi city on 64 male esophageal cancer patients used the subjective global assessment (SGA) score to determine 50.2% of patients in the malnutrition class (class B and C) [18].

Cancer and nutritional status have a strong relationship, such as overweight and obesity, which may increase the risks of several types of cancer [19,20,21]. Cancer-associated malnutrition poses significant risks to the patients as it results in many consequences such as loss of appetite, loss of body weight, alterations in body composition, and decline in physical functions [12,22]. Malnutrition in cancer patients is also associated with adverse consequences such as longer hospital stay, lower tolerance of cancer treatment, and reduction of quality of life [23,24]. A study in China reported patients without the need of nutritional supports had significantly higher survival time than those in need of these supports [25]. Furthermore, malnutrition also leads to severe impairments in hepatic peroxisomal and mitochondrial function and hepatic metabolic dysfunction [26], and is involved in important decreases in essential fatty acids in very low density lipoproteins, such as triacylglycerols and phospholipids [27]. For esophageal patients, those who received nutritional interventions had a lower mean of length of hospital stay, as well as hospital charges, than those who did not [28,29]. Along with surgery and other non-pharmacological treatments, nutritional support has been proven to improve the treatment effect for cancer, resulting in quality of life improvement and positive long-term outcomes for patients [7].

Providing adequate and appropriate nutrition support for patients with gastrointestinal surgery is an important and urgent task. Previous study have found that there was an association between nonalcoholic fatty liver disease (NAFLD) and esophagus cancer [30]. A healthy and balanced diet could help decrease the risk and improve the treatment of both NAFLD and esophagus cancer [31,32].

The Vietnam National Cancer Hospital is a leading hospital in the country for the diagnosis and treatment of cancer diseases. This study aims to assess the nutritional status and feeding regime of newly diagnosed esophageal cancer patients in Vietnam National Cancer Hospital from 2017 to 2018. Based on that, measures to improve the quality of care and treatment for patients with esophageal cancer are proposed to reduce complications and medical costs, as well as the length of hospital stay, for patients with nutritional problems.

## 2. Methods

### 2.1. Participants and Study Design

This was a cross-sectional study on patients of Vietnam National Cancer Hospital (NCH) with esophageal cancer diagnoses from September 2017 to June 2018. The NCH is a national oncology hospital that treats many referral cancer cases in the north of Vietnam. In the country, most esophageal cancer patients are at stage III/IV and receive percutaneous endoscopic gastrostomy (PEG) before chemo- or radiotherapy. Thus, after the hospital admission, the patients usually go to the surgery department for the PEG. This study recruited newly diagnosed esophageal cancer patients who had indicators for PEG but did not have any tumor treatments before or comorbidities that could affect nutritional status (such as gastrointestinal disease or chronic kidney disease), and had stayed more than 7 days in the hospital. This research was approved by the scientific committee of the Hanoi Medical University (approval no.5076/QĐ-ĐHYHN) on 8 August 2017. All patients participated voluntarily and signed the informed consent forms.

### 2.2. Data Collection

When patients were admitted to the hospital, they were interviewed, and measurements were made of anthropometrics, such as weight, height, and mid upper arm circumference (MUAC). The nutritional status was assessed by Patient-Generated Subjective Global Assessment (PG-SGA), 24 h dietary intake before surgery, albumin, and hemoglobin blood before surgery. After surgery, the patients were weighed again, and their feeding regimes and blood pre-albumin were investigated within 7 days.

### 2.3. BMI Calculation

The BMI was calculated using the weight of patients, expressed in kilograms divided by the height of patients, expressed in meters squared (kg/m^2^). The World Health Organization (WHO) criteria [33] were used to determine the nutritional status of patients:

- BMI ≥ 25: overweight/obese
- 18.5–24.99: normal
- CED level 1: 17–18.49 (mild chronic energy deficiency (CED)).
- CED level 2: 16–16.99 (moderate CED).
- CED level 3: <16.0 (severe CED).

### 2.4. Measurement of Mid Upper Arm Circumference (MUAC)

The left MUAC of the patients was measured with the cut-off points of 22 and 23 cm for women and men, respectively [34]. The decision to use MUAC over other anthropometric indices and measures was based on a systematic review in 2016, reporting that MUAC is a simple, acceptable, reliable, and cost-effective measure to assess the nutritional status of patients [35].

### 2.5. Patient-Generated Subjective Global Assessment

All patients were assessed by PG-SGA scores that consisted of 3 sections. The first section, which was completed by the patients, comprised the following components: weight, food intake, symptoms, and activities and function. The physician completed the professional component part including metabolic stress, physical examination, nutritional requirements, and weight loss scoring. In the global assessment section, the PG-SGA scores were categorized as [36]:

+ A: Well nourished.
+ B: Mild/moderately malnourished.
+ C: Severely malnourished.

➢ *Serum albumin*: in adults.
  + Normal: 35–50 g/L
  + Mildly undernourished: 28 – < 35 g/L.
  + Moderately undernourished: 21 – < 28 g/L.
  + Severely undernourished: <21 g/L.
➢ *Serum prealbumin*:
  + Normal: 20–40 mg/dL
  + Mildly undernourished: 17 – < 20 mg/dL
  + Moderately undernourished10 – < 17 mg/dL
  + Severely undernourished < 10 mg/L [37].
➢ Total lymphocyte count—TLC:
  + Normal: >1800/mm^3^
  + Mild malnutrition: 1500–1800/mm^3^
  + Moderate malnutrition: 900 – < 1500/mm^3^
  + Severe malnutrition: <900 mm^3^.
➢ *Hemoglobin*: diagnosis anemia when hemoglobin was <130 g/L in men and was <120 g/L in women.
➢ *24 h dietary recall*:

The dietitian administered the 24 h diet of the patients. The method of recall was described elsewhere [18].

### 2.6. Data Analysis

All input data was statistically analyzed using STATA v12.0 for MacBook (Stata Corp., College Station, TX, USA). A t-test was used to compare 2 mean values with delimitation standards, and an ANOVA test was used to compare multiple mean values with the significance level *p* < 0.05. For categorical or binary variables, the Phi/Cramer correlation coefficient was used.

## 3. Results

All patients in our study were male. The study showed that the patients’ age ranged from 38 to 88 years with the most common range of 40 to 59 years (58.5%) and the mean age of 57.1 ± 8.5 years. There was a small number of poor patients (8.3%) and near-poor patients (7.8%) based on socio-economic status. Most patients were in stage III (60.2%) (Table 1).

Table 2 describes the characteristics related to the nutritional status of patients. The patients’ pre-operation and 1-week-post-operation mean weight was 50.2 and 49.5 kg, respectively. The average BMI was 18.8 ± 2.8 kg/m^2^ and the average MUAC was 23.6 ± 2.6 cm. The mean of biochemical indicators was albumin 39.7 ± 4.1 g/L; prealbumin was 15.7 ± 5.4 mg/dL; TLC was 2.4 ± 0.9; and hemoglobin was 133.7 ± 16.4 g/L (Table 2).

The BMI data revealed that 47.6% of patients were underweight, whilst the MUAC indicated 50% of patients were undernourished. Using evaluation of nutritional status by PG-SGA, the study showed that 52.9% of patients suffered from mild/moderate malnutrition, and 29.6% of them had severe malnutrition. Our results showed that dysphagia was the most common symptom of esophageal cancer in patients, accounting for 87.4%. Moreover, 63.1%, 82.5%, and 90.8% of patients reported weight loss one week after surgery, and one month and six months pre-diagnosis, respectively. The proportions of patients having malnutrition were 10.7%, 55.8%, and 27.2% according to albumin, prealbumin, and total lymphocyte count, respectively. Moreover, 34.5% of esophageal cancer patients suffered from anemia (Table 2).

Table 3 shows the association between percentage of weight loss over the past 1 month and digestive symptoms. Specifically, patients with dysphagia were at 3.39 times higher risk than those without dysphagia to lose >10% of their body weight (*p* < 0.05). An approximately similar OR was observed in the group of patients who frequently felt painful blockage compared to those who did not. Additionally, the study found that patients reporting fatigue and anorexia symptoms were much more likely to lose >5% of bodyweight than asymptomatic patients (*p* < 0.05).

The BMI value had a negative correlation with the PG-SGA (r = −0.212, *p* = 0.001). Both BMI and PG-SGA were negatively correlated with the albumin (r = −0.936, *p* < 0.05 and r = −0.563, *p* < 0.05, respectively). The PG-SGA revealed a correlation with prealbumin (r = 0.676, *p* = 0.042) (Table 4).

The means of energy, protein, lipid, and carbohydrate values of the 24 h preoperative diets of patients were 973.6 ± 443.0 kcal/day, 42.4 ± 21.6 g/day, 31.0 ± 15.5 g/day, and 130.0 ± 64.5 g/day, respectively (Table 5).

On the first day of feeding, the energy was supplied intravenously to the patients with an average energy of 631.6 ± 358.5 kcal. From the second day onwards, more energy intake came from the gastrointestinal tract but included both oral and nasal feeding tubes. The oral energy intake of the patients increased over the days, while the energy intake from the nasal feeding tube increased to the highest on the fourth day to 548.9 ± 243.7 kcal, and then decreased over the following days. Total energy intake increased day by day, with the average energy intake of 1343.9 ± 521.3 kcal on the seventh day (Table 6).

The dietary proportions of patients achieving the European Society for Clinical Nutrition and Metabolism (ESPEN)’s energy and protein recommendations were very low. On the first day after surgery, none of the patients met the need for energy. Each day, ESPEN’s recommended energy demand was increased and reached the highest level on the seventh day after surgery (18.0%). In terms of protein, on the first day after surgery, 1.5% of the patients achieved the recommended dose. Protein response rates increased day by day and were highest at 7 days post-operation at 19.4% (Table 7).

The postoperative diet for patients provided an inadequate vitamin intake (Table 8). Diet frequency only fulfilled the general recommendation for a few vitamins. This frequency increased in the days after surgery when the patients were fed orally, while on the first day after surgery, the patient was completely nourished intravenously with no vitamins or minerals added. In our study, most patients were not fed with enough energy and protein, and none of them received any vitamin supplements. These factors could have affected the wound healing process of patients, and hence attention should be given to ensure the adequate nutrition for patients after surgery.

## 4. Discussion

Our study of 206 patients with esophagus cancer showed that the patients’ ages ranged from 38 to 88 years with the most common range being 40 to 59 years (58.5%), and the mean age was 57.1 ± 8.5 years. Only one patient was under the age of 40 (Table 1). This result was consistent with the epidemiology of this disease, as it commonly emerged in the age group of 50 to 60 years. However, this was lower in comparison with international studies. According to Launoy et al. [38], the average age of male patients was 65 years old. The difference in average age with international studies might arise from the lower life expectancy of Vietnamese people than in developed countries. Most patients were in stage III (60.2%), stage II accounted for 22.3%, and the figures for stage I and IV were 7.8% and 9.7%, respectively. Our results were similar to those of studies in America and China, with 56.0% and 60.8% of patients diagnosed with esophagus cancer stages III and IV [39,40]. Vietnam is still a developing country, and due to limited economic conditions and people’s limited awareness of cancer, patients are often not examined when early manifestations of cancer occur, and their health is only checked when the disease has become intolerable.

In evaluating nutritional status by PG-SGA, the study found that 52.9% of patients suffered from mild/moderate malnutrition, and 29.6% had severe malnutrition. Compared with other world studies, the results showed that there were differences in nutritional risk between countries and regions. A study by Poziomyck et al. in patients with gastrointestinal tumors showed that 66.2% of patients were malnourished (SGA B: 45.9% and SGA C: 20.3%) [41]. A study evaluating the nutritional status of gastrointestinal cancer patients in China found that there were 44.2% of patients with mild to moderate risk, and 4% of patients with severe risk of malnutrition according to SGA [42]. Another study by Faramarzi et al. in Iran showed that malnutrition prevalence was 52%, and concluded PG-SGA was a useful tool in the screening of malnutrition in cancer patients [43].

Our results shows that dysphagia is the most common symptom of esophagus cancer patients, accounting for 87.4%. This result was quite similar to other world studies. Hamrah’s study in Afghanistan found that 84.8% of esophagus patients suffered from dysphagia [44]; another study by Ripley et al. found that 53.5% of patients had a difficult degree of dysphagia [39], while this figure in a study by Wu et al. in China was significantly high at 92.9% [45]. Dysphagia made patients afraid to eat, making the nutritional status of the patients worse. The anorexia prevalence in our study was 28.2%. Other studies found that anorexia was one of the most common complications in cancer patients, with 24% being anorexic at the time of diagnosis, 80% being anorexic in the advanced stage, and 66% being anorexic when undergoing chemotherapy [46,47]. Anorexia develops rapidly and recovery is difficult, hence early intervention is necessary for the patient’s nutritional status [48,49].

Our results show that more than 80% of the cancer patients lost weight in the past one and six months. Based on the percentage of their bodyweight loss, these patients had entered the stage of pre-cachexia or cachexia stages [50]. These results are consistent with the evidence that up to 85% of esophageal patients experienced weight loss before undergoing surgery [51] and up to 80% of cancer patients in general experienced the symptoms and signs of cachexia including weight loss, anorexia, fatigue and anemia, and metabolism disorders [50,52].

The results of our research are in line with many studies showing that weight loss is very common among cancer patients, especially those with gastrointestinal cancer. A study by Nhung (2015) reported the percentages of patients losing their bodyweight over the past one and six months were 35.3% and 68.7%, respectively [53]. Another study by van der Schaaf et al. showed that 100% of study patients with esophageal or gastroesophageal cancer suffered from weight loss [17]. A study in China also reported that only 18.5% of patients with esophageal squamous cell carcinoma experienced no weight loss during their treatment [54].

Additionally, it was reported that up to 63% of cancer patients under chemotherapy treatment lost their weight at different severities [55]. A Malaysian study found that about one third of patients lost >5% of their weight within the first month after diagnosis and continuous weight loss was shown to be a robust predictor of cancer complications and a underlying cause of malnutrition [56]. Therefore, it is evident that weight loss is one of the important factors that need to be controlled in cancer patients and one of the parameters that need to be included in the nutritional status assessment and monitoring tools to improve the nutritional status and reduce poor prognosis and complications for cancer patients.

Our study also found the association between weight loss over the past month and digestive symptoms, and the results were comparable to a Mexican study, which also showed that cancer patients suffering from nausea, vomiting, and anorexia had significantly higher risk of losing >10% of bodyweight [55]. Therefore, it can be seen that digestive symptoms, especially anorexia, loss of appetite, and early satiety, substantially affects the food intake, leading to the severe calorie deficit. If all the fat is burned for energy, the body has to use the protein from muscle for its fuel, leading to muscle atrophy and malnutrition. In addition, vomiting and diarrhea also resulted in weight loss by causing the water and electrolyte imbalance, which decreases volume circulation and increases unabsorbed nutrients.

Our study showed that the average weight of patients before surgery was 50.2 kg, and 7 days after surgery, the patients’ average weight dropped to 49.5 kg. On average, weight decreased by 0.7 kg at 7 days after surgery. The patient’s weight loss might be due to inadequate postoperative care, which was insufficient according to recommended needs. Compared to other studies in the world, patients in our study lost less weight. Beattie et al.’s study of surgical patients (mainly gastrointestinal surgery) showed that 2 weeks after surgery, the weight of the patients dropped by 4.21 kg [57]. Similarly, Lopes et al. also showed a decrease in weight in patients after surgery with preoperative and preoperative mean weights of 74.1 and 70.4 kg, respectively [58]. This difference was due to the gastric opening surgery being uncomplicated, so the patient lost less fluid and blood than the surgeries in the above studies. Weight loss was a predictor of decreased survival rate in cancer patients and associated with decreased body function, anxiety, and poor quality of life [58]. Postoperative weight loss and weight loss prior to surgery would exacerbate the problem, causing adverse effects to the patient. Poorer outcomes in patients undergoing gastrointestinal tract surgery and suffering from weight loss would reduce the patients’ tolerance to adjuvant therapy, and increased the rate of emerging chemotherapy toxicity [59,60,61,62] and surgical complications [62,63]. Therefore, attention should be paid to nutritional support to improve the nutritional status of patients after surgery.

The patients’ average preoperative BMI was 18.8 kg/m^2^. This result was lower than that of Quyen et al. (19.9 kg/m^2^) [18]. The subject of that study was esophagus cancer patients being treated with chemotherapy or radiation therapy, causing the symptoms of dysphagia to slightly decrease, so the patients could eat better. The average BMI of esophagus cancer patients in a study by Wu et al. in China was 21.6 kg/m^2^ [45], and the average BMI of such patients in a study by Di Fiore et al. in France was 22.5 kg/m^2^ [64]. This could be due to the small stature of Vietnamese people, compared to Chinese and French people. Apart from that, Vietnamese patients often only detected the disease in its late stages, so the symptoms such as dysphagia, anorexia, fatigue, etc. significantly affected the nutritional status of the patients.

In comparison with PG-SGA, the proportions of malnourished patients according to BMI were significantly lower than that of the PG-SGA (47.6% vs. 81.5%). This could be explained by the fact that malnutrition is a dynamic process, accompanied by steady weight loss even in overweight people. In addition, the clinical examination index included three symptoms: reduced subcutaneous fat, signs of fluid retention, and reduced muscle mass. These symptoms, if based solely on BMI, were often insignificant, so a person might be normal according to BMI but malnourished based on PG-SGA. Many patients with malnutrition risk might be missed. Therefore, the threshold of 18.5% was far from perfect, and should not be used as a sole indicator for evaluating nutritional status [65]. Meanwhile, the PG-SGA tool kit was used in addition to the use of anthropometric measures while weighing up the weight loss for the past 1 and 6 months, with the emergence of varying symptoms in the diet and clinical signs evaluation. Therefore, it is better to use anthropometry with other indicators/signs (especially weight loss and changes in diet) to correctly and comprehensively evaluate the nutritional status of these patients.

Serological albumin is not a good indicator of malnourishment status as it is less sensitive than clinical examinations and medical history interviews [66]. The evaluation of the preoperative albumin index showed that the majority of patients were free from malnourishment (89.3%). The incidence of mild and moderate malnutrition was 9.2% and 1.5%, respectively, and no patients had severe malnutrition. The results were significantly lower than the BMI and PG-SGA scores as they showed 47.6% and 82.5% of patients at risk of malnutrition, respectively. The results indicated that if only the albumin index was used to assess the nutritional status of cancer patients, fewer patients would be at risk of malnutrition. This was because albumin had a long half-life of 18–20 days, and it was also affected by liver function and some other factors, such as some patients having severe clinical signs of malnourishment (muscle atrophy, subcutaneous fat loss, weight loss, poor diet, etc.). However, with reduced circulation volume due to fluid loss, albumin could increase despite the fact that patients were suffering from severe malnourishment.

The pre-albumin has a much shorter half-life than albumin (two days). It is, therefore, more sensitive to protein–energy changes than albumin [67]. Pre-albumin levels reflect a recent diet rather than overall nutritional status. Pre-albumin levels are expected to be a useful marker of nutritional status and are used to help detect and diagnose malnourishment and nutritional deficiencies, as well as monitoring nutritional intake. The mean pre-albumin value was 18.5 mg/dL. This result was lower than in other studies. The average pre-albumin in esophagus cancer patients was 22.0 mg/dL in Saudi Arabia [68], and 21.7 mg/dL in China [45]. Those studies were conducted on patients one year after esophageal surgery [68] and non-weight loss cancer patients [45].

Our hemoglobin results were on par with some other world studies—36% in a study in Japan [69], 31% in a study in India [70], and 29.1% in a study in China [71]. As such, it could be seen in most studies that the proportion of patients suffering from anemia was very high. Therefore, nutrition counseling for cancer patients should not only focus on the supplementation of nutrient-rich and high energy foods, but also focus on iron-rich foods to help stimulate red and other blood cells.

The 24 h preoperative diet of patients had an average energy intake of 973.6 kcal/person/day, protein intake of 42.4 g/person/day, lipid intake of 31.0 g/person/day, and glucide intake of 130.0 g/person/day. Only nine patients had sufficient diets (4.37%). This could be due to severe symptoms of the disease (dysphagia, pain, fatigue, anorexia, vomiting, nausea, etc.) preventing the patients from eating. Apart from that, the main diet of the patients was porridge and milk, and some even could only drink milk, so the daily intake was insignificant. This was one of the reasons that the 24 h preoperative diet did not provide enough energy and micronutrients for patients. When comparing a 24 h preoperative diet based on socio-economic status, poor patients’ diet had lower total energy, total protein, animal protein, total lipid, plant lipid, and glucide than those of the other patients. This was because poor patients usually came from poor rural areas and could not afford to pay for transport and medical expenses, not to mention spending on nutritious meals. Moreover, Vietnam National Cancer Hospital has a high number of daily charity meals. This group of patients was more likely to choose charity meals to fill their plate than to pay for a nutrient-rich diet. Diets among PG-SGA classification groups had an increasing intake of energy, total protein, animal protein, total lipid, plant lipid, and glucose as assessed by PG-SGA levels A, B, and C. PG-SGA was a subjective and comprehensive assessment performed on all aspects, including diet and symptoms affecting the patient’s diet, such as anorexia, dysphagia, vomiting, nausea, fatigue, etc. Furthermore, an unbalanced diet could increase the risk of getting diseases that the mechanisms and therapeutical approach for are still debatable, such as esophagus cancer or NAFLD [72].

On the first day of feeding, the energy was supplied intravenously to the patient with the average energy of 631.6 ± 358.5 kcal. The result illustrated the lack of intravenous feeding. The health insurance policies for paid intravenous products had restricted doctors in specifying this to patients. From the second day onwards, more energy intake came from the gastrointestinal tract, but included both oral and nasal feeding tubes. The oral energy intake of the patients increased over the days, while the energy intake from the nasal feeding tube increased to the highest on the fourth day to 548.9 ± 243.7 kcal, and then decreased over the following days. Total energy intake increased day by day, with the average energy intake of 1343.9 ± 521.3 kcal in the seventh day. Those results were higher than those of Quyen’s study with only 1208 kcal/day [18]. The postoperative diet for patients provided an inadequate vitamin intake. Diet frequency only fulfilled the general recommendation for a few vitamins. On the first day after surgery, the patient was completely nourished intravenously with no vitamins and minerals added. After that, the patients were fed orally, so the frequency of patients with an inadequate vitamin intake increased.

There are several limitations of our study. The cross-sectional design of this study meant that any potential associations we found did not warrant causal relationship. Our study was also subject to recall bias as the patients had to remember their diet in the past month and 24 h before undergoing PEG. Furthermore, we were unable to access the specific diet of each patient recommended by the hospital’s nutritionist before the surgery. Therefore, we had to use the 24 h dietary recall before the surgery. Finally, as there is a proven association between NAFLD and gastrointestinal tract cancer, we did not have data on the NAFLD prevalence amongst our study’s participants to highlight this association.

## 5. Conclusions

Malnutrition and insufficient energy intake were substantially noticeable in patients suffering from esophagus cancer. The PG-SGA score was found to be correlated with the dietary intake of patients (total energy, total protein, animal protein, total lipid, and vegetable lipid). In this study, most patients were not fed with enough energy and protein, and none of them received any vitamin supplements. These factors could affect the wound healing process of patients. Hence, attention should be given to ensure adequate nutrition for patients after surgery. Furthermore, public awareness should be raised on the importance of recognizing weight loss as an early symptom of esophageal cancer and all cancer types in general and the utilization of in-depth preoperative assessment tools for assessing nutritional status and managing malnutrition.

## Figures and Tables

**Table 1:** Patient characteristics.

Patient Characteristics		*n*	%
Age (years)	40–59	121	58.7
	18–39 and ≥60	85	41.4
	Average	57.1 ± 8.5	
Sex	Male	206	100.0
Socio-economic status	Poor	17	8.3
	Near poor	16	7.8
	Normal	173	83.9
Tumor location	Upper	44	21.4
	Middle	93	45.1
	Lower	69	33.5
Stage	Stage 0 and I	16	7.8
	Stage II	46	22.3
	Stage III	124	60.2
	Stage IV	20	9.7
Tumor type	Adenocarcinoma	200	97.1
	Squamous cell and others	6	2.9

**Table 2:** Characteristics on anthropometric measurements, Patient-Generated Subjective Global Assessment (PG-SGA), weight change, symptoms of digestive system, and biochemical indicators.

Characteristics		*n* (%)	Mean ± Standard Deviation (Range)	Median
Anthropometric Measurements				
*Weight (kg)*	Pre-operation Post-operation		50.2 ± 8.2 (28.6–76.6) 49.5 ± 8.5 (30.6–75.9)	49.2 53.5
*Height (cm)*			163.3 ± 5.9 (147–176)	163.8
*Body mass index (kg/m^2^)*	<18.5 18.5–24.9 ≥25	98 (47.6) 102 (49.5) 6 (2.9)	18.8 ± 2.8 (10.5–28.1)	18.5
*Mid upper arm circumference (cm)*	Undernutrition Normal	103 (50.0) 103 (50.0)	23.6 ± 2.6 (16.8–36.0)	23.8
*PG-SGA*	PG-SGA A PG-SGA B PG-SGA C	36 (17.5) 109 (52.9) 61 (29.6)		
*Symptoms of digestive system*	Dysphagia Pain Fatigue Anorexia Constipation	180 (87.4) 105 (51.0) 102 (49.5) 58 (28.2) 44 (21.4)		
*Weight change*				
Weight change past 1 week	Weight loss Weight stable Weight gain	130 (63.1) 6 (2.9) 70 (34.0)		
Weight change past 1 month	Weight loss Weight stable Weight gain	170 (82.5) 14 (6.8) 22 (10.7)		
Weight change past 6 months	Weight loss Weight stable Weight gain	187 (90.8) 9 (4.4) 10 (4.9)		
*Albumin (g/L)*	Normal Mildly undernourished Moderately undernourished Severely undernourished	184 (89.3) 19 (9.2) 3 (1.5) 0 (0)	39.7 ± 4.1 (27–47.8)	40
*Prealbumin (mg/dL)*	Normal Mild malnutrition Moderate malnutrition Severe malnutrition	91 (44.2) 50 (24.3) 41 (19.9) 24 (11.7)	15.7 ± 5.4 (2–32)	16
*Total lymphocyte count*	Normal Mild malnutrition Moderate malnutrition Severe malnutrition	150 (72.8) 28 (13.6) 22 (10.7) 6 (2.9)	2.4 ± 0.9 (0.6–9.1)	2.3
*Hemoglobin*	Normal Anemia	135 (65.5) 71 (34.5)	133.7 ± 16.4 (76–189)	135

**Table 3:** The association between percentage of weight change in the past 1 month and digestive symptoms.

Symptoms	% of Weight Change Past 1 Month			
	>5%		>10%	
	OR (95% CI)	*p*	OR (95% CI)	*p*
Dysphagia	1.75 (0.9–3.6)	0.11	**3.39 (1.0–11.6)**	**0.04**
Pain	1.59 (0.7–3.5)	0.24	**3.5 (1.0–12.0)**	**0.037**
Fatigue	**3.1 (1.5–6.5)**	**0.001**	2.96 (0.86–10.2)	0.07
Anorexia	**2.2 (1.1–4.5)**	**0.025**	1.9 (0.5–6.7)	0.3
Constipation	1.2 (0.6–2.6)	0.59	1.85 (0.5–6.6)	0.34

Note: *p* values were determined by chi-squared test (*p* < 0.05).

**Table 4:** The correlation between PG-SGA, body mass index (BMI), and biomarkers.

		BMI	Albumin	Prealbumin	Total Lymphocyte Count	Hemoglobin
PG-SGA	r	−0.212 **	−0.563 *	0.676 *	−0.834	−0.289
	*p*	0.001	0.03	0.042	0.066	0.072
BMI	r		−0.936 *	0.987	−0.643	−0.746
	*p*		0.028	0.055	0.057	0.065
Albumin	r	−0.936 *		0.436	−0.689	0.738
	*p*	0.028		0.048	0.121	0.436
Prealbumin	r	0.987	0.436		0.639	0.822
	*p*	0.055	0.048		0.067	0.236
Total Lymphocyte Count	r	−0.643	−0.689	0.639		−0.569
	*p*	0.057	0.121	0.067		0.73
Hemoglobin	r	−0.746	0.738	0.822	−0.569	
	*p*	0.065	0.436	0.236	0.73	

* Correlation is significant at the 0.05 level (2-tailed). ** Correlation is significant at the 0.001 level (2-tailed).

**Table 5:** Dietary intake in 24 h recall before surgery.

Characteristics		Energy kcal/24 h	Protein (g/24 h)		Lipid (g/24 h)		Carbohydrate (g/24 h)
			Total Protein	Animal Protein	Total Lipid	Plant Lipid	
^a^ Age (years)	<60 (n = 122)	998.2 ± 432.6	43.4 ± 21.5	27.1 ± 15.1	31.1 ± 13.9	12.7 ± 7.7	135.2 ± 65.9
	≥60 (n = 84)	937.7 ± 457.8	41.0 ± 21.7	27.0 ± 15.9	30.9 ± 17.6	12.5 ± 8.1	122.5 ± 62.2
^a^ Socio-economic status	Poor or near poor (n = 33)	770.5 ± 400.8 *	34.1 ± 21.2 *	20.2 ± 13.7 *	25.6 ± 15.0 *	9.9 ± 7.1 *	100.4 ± 51.7 *
	Normal (n = 173)	1012.3 ± 441.1	44.0 ± 21.3	28.3 ± 15.4	32.1 ± 15.4	13.1 ± 7.9	135.7 ± 65.3
^b^ PG-SGA	PG-SGA A (n = 36)	1067.4 ± 363.7 *	49.0 ± 18.9 *	29.0 ± 12.7 *	31.9 ± 11.2 *	16.1 ± 7.4 *	145.8 ± 58.9 *
	PG-SGA B (n = 109)	1066.2 ± 435.6	46.2 ± 21.3	30.2 ± 14.6	33.7 ± 15.2	14.1 ± 6.9	143.5 ± 64.1
	PG-SGA C (n = 61)	752.7 ± 425.2	31.7 ± 20.0	20.3 ± 16.3	25.7 ± 17.0	7.9 ± 7.7	96.7 ± 56.4
Total		973.6 ± 443.0	42.4 ± 21.6	27.0 ± 15.4	31.0 ± 15.5	12.6 ± 7.9	130.0 ± 64.5

*: *p* < 0.05, ^a^: *p* values were determined by *t*-test; ^b^: *p* values were determined by ANOVA. When comparing the energy values by age groups (over 60 years vs. under 60 years), the socio-economic classification and PG-SGA studies showed that there was a difference between groups. However, only a few groups had a statistically significant difference. That was, the relationship between the poor or near-poor patients and total energy, total protein, animal protein, total lipid, plant lipid, and carbohydrate was lower than these normal socio-economic patient groups. The percentage distribution among groups of patients classified by PG-SGA with energy, total protein, animal protein, total lipid, plant lipid, and carbohydrate was statistically different with *p* < 0.05 (Table 5).

**Table 6:** Average postoperative (D1–D7) dietary intake according to energy source (parenteral, enteral, oral).

Day	Total Parenteral Nutrition (kcal)	Oral Nutrition (kcal)	Enteral Nutrition (Tube Feeding) (kcal)	Total (kcal)
Day 1	631.6 ± 358.5	0	0	631.6 ± 358.5
Day 2	370.2 ± 213.0	139.6 ± 95.7	129.6 ± 68.9	650.2 ± 392.2
Day 3	305.5 ± 201.4	246.5 ± 103.8	369.6 ± 156.9	941.5 ± 474.4
Day 4	301.8 ± 237.7	420.1 ± 176.3	548.9 ± 243.7	1070.1 ± 401.3
Day 5	275.9 ± 189.7	532.8 ± 256.4	478.6 ± 279.6	1121.5 ± 432.6
Day 6	165.9 ± 79.8	996.9 ± 420.0	254.9 ± 156.4	1206.6 ± 456.2
Day 7	165.9 ± 79.8	1021.7 ± 368.9	274.6 ± 164.7	1343.9 ± 521.3

**Table 7:** Comparison of average postoperative (D1–D7) dietary intake according to European Society for Clinical Nutrition and Metabolism (ESPEN).

Day		Energy		Protein	
		n	%	n	%
Day1	Not reached	206	100	203	98.5
	Reached	0	0	3	1.5
Day 2	Not reached	203	98.5	198	96.1
	Reached	3	1.5	8	3.9
Day 3	Not reached	196	95.1	186	90.3
	Reached	10	4.9	20	9.7
Day 4	Not reached	194	94.2	183	88.8
	Reached	12	5.8	23	11.2
Day 5	Not reached	191	92.7	179	86.9
	Reached	15	7.3	27	13.1
Day 6	Not reached	185	89.8	174	84.5
	Reached	21	10.2	32	15.5
Day 7	Not reached	169	82.0	166	80.6
	Reached	37	18.0	40	19.4

**Table 8:** Postoperative feeding regime for some vitamins and minerals of esophageal cancer patients.

	Day 1 (Mean; Range)	Day 2 (Mean; Range)	Day 3 (Mean; Range)	Day 4 (Mean; Range)	Day 5 (Mean; Range)	Day 6 (Mean; Range)	Day 7 (Mean; Range)
Vitamin B1 (mg)	0	0.4; 0–2.8	0.9; 0–4.2	1.2; 0–3.9	1.3; 0–4.1	1.0; 0–4.5	1.1; 0–3.8
Vitamin B2 (mg)	0	0.3; 0–1.6	0.6; 0–3.3	1.1; 0–4.3	0.8; 0–3.9	0.9; 0–4.2	1.1; 0–4.4
Vitamin PP (mg)	0	0.5; 0–3.2	0.9; 0–3.6	1.5; 0–6.3	1.7; 0–6.6	2.1; 0–8.6	2.4; 0–10.3
Vitamin B6 (mg)	0	0.3; 0–2.1	0.7; 0–3.1	1.2; 0–4.5	1.0; 0–4.3	1.2; 0–4.6	1.5; 0–5.1
Folate (µg)	0	33.5; 0–231	76.7; 0–351	102.9; 0–344	134.6; 0–367	167.9; 0–394	156.7; 0–356
Vitamin B12 (µg)	0	0.9; 0–12	1.9; 0–12.8	2.5; 0–13.4	2.6; 0–13.2	2.9; 0–12.5	2.7; 0–13.9
Vitamin C (mg)	0	29.6; 0–279	70.1; 0–378.8	89.1; 0–514.4	91.6; 0–498.7	85.7; 0–501.7	87.4; 0–562.3
Vitamin A (µg)	0	72.6; 0–892.1	159.4; 0–1032	209.9; 0–1455	198.6; 0–1342	207.6; 0–1508	212.6; 0–1543
Vitamin D (µg)	0	1.7; 0–19.4	3.5; 0–19.6	4.4; 0–22.7	4.5; 0–23.6	3.9; 0–21.5	3.7; 0–25.4
Vitamin E (mg)	0	2.6; 0–19.8	5.9; 0–24.7	7.7; 0–23.5	6.8; 0–25.8	7.2; 0–28.7	7.5; 0–21.6
Vitamin K (µg)	0	12.9; 0–282.5	32.1; 0–776.7	47.4; 0–776.7	48.9; 0–789.7	49.6; 0–725.8	51.2; 0–698.3
Calcium (mg)	0	133.6; 0–864	308.9; 0–2127.5	401.2; 0–2129	434.5; 0–1987	437.6; 0–2065	452.4; 0–2012
Iron (mg)	0	1.5; 0–10.8	3.6; 0–16.5	4.8; 0–12.9	3.9; 0–13.6	4.2; 0–11.9	4.5; 0–15.8
Phosphorus (mg)	0	127.7; 0–784.2	320.6; 0–1600.5	435.6; 0–1600.5	389.7; 0–1534.1	402.7; 0–1600.5	406.9; 0–1524.9
Zinc (mg)	0	2.3; 0–20.8	5.2; 0–25.4	6.9; 0–23.4	6.2; 0–21.9	5.8; 0–19.8	5.3; 0–23.4

## Data Availability

Data sharing not applicable. No new data were created or analyzed in this study. Data sharing is not applicable to this article.

## Abbreviation

BMI	Body mass index
CED	Chronic energy deficiency
DALYs	Disability-adjusted life years
ESPEN	The European Society for Clinical Nutrition and Metabolism
MUAC	Mid upper arm circumference
NCH	Vietnam National Cancer Hospital
PEG	Percutaneous endoscopic gastrostomy
PG-SGA	Patient-Generated Subjective Global Assessment
SGA	Subjective Global Assessment
WHO	World Health Organization
